# Oral Administration of *Lactobacillus helveticus* LA401 and *Lactobacillus gasseri* LA806 Combination Attenuates Oesophageal and Gastrointestinal Candidiasis and Consequent Gut Inflammation in Mice

**DOI:** 10.3390/jof7010057

**Published:** 2021-01-15

**Authors:** Hélène Authier, Marie Salon, Mouna Rahabi, Bénédicte Bertrand, Claude Blondeau, Sarah Kuylle, Sophie Holowacz, Agnès Coste

**Affiliations:** 1 UMR 152 Pharma-Dev, Université de Toulouse, IRD, UPS, 31432 Toulouse, France; marie.salon@wanadoo.fr (M.S.); mounarahabi@yahoo.com (M.R.); benedicte.bertrand1@univ-tlse3.fr (B.B.); 2 PiLeJe Laboratoire, 75015 Paris, France; c.blondeau@pileje.com (C.B.); s.holowacz@pileje.com (S.H.); 3 GENIBIO, 91290 Lorp-Sentaraille, France; sarah.kuylle@gmail.com

**Keywords:** gastrointestinal candidiasis, gut inflammation, macrophages, *Lactobacillus gasseri*, *Lactobacillus helveticus*, probiotic, C-type lectin receptor, dectin-1, mannose receptor

## Abstract

*Candida albicans* is an opportunistic pathogen that causes mucosal gastrointestinal (GI) candidiasis tightly associated with gut inflammatory status. The emergence of drug resistance, the side effects of currently available antifungals and the high frequency of recurrent candidiasis indicate that new and improved therapeutics are needed. Probiotics have been suggested as a useful alternative for the management of candidiasis. We demonstrated that oral administration of *Lactobacillus gasseri* LA806 alone or combined with *Lactobacillus helveticus* LA401 in *Candida albicans*-infected mice decrease the *Candida* colonization of the oesophageal and GI tract, highlighting a protective role for these strains in *C. albicans* colonization. Interestingly, the probiotic combination significantly modulates the composition of gut microbiota towards a protective profile and consequently dampens inflammatory and oxidative status in the colon. Moreover, we showed that *L. helveticus* LA401 and/or *L. gasseri* LA806 orient macrophages towards a fungicidal phenotype characterized by a C-type lectin receptors signature composed of Dectin-1 and Mannose receptor. Our findings suggest that the use of the LA401 and LA806 combination might be a promising strategy to manage GI candidiasis and the inflammation it causes by inducing the intrinsic antifungal activities of macrophages. Thus, the probiotic combination is a good candidate for managing GI candidiasis by inducing fungicidal functions in macrophages while preserving the GI integrity by modulating the microbiota and inflammation.

## 1. Introduction

*Candidaalbicans* is both an opportunistic fungal pathogen and a normal member of the gastrointestinal microbiota adapted to colonize all segments of the digestive tract from the oral cavity to the anus [1,2]. *C. albicans* exists in harmony with other microorganisms of the microbiota in most individuals with a healthy immune system [1]. However, dysbiosis resulting, for example, from variations in the local environment (pH shifts or nutritional changes), antibiotic treatment, or alterations in the immune system can favor *C. albicans* rapid proliferation and cause infections. These infections range from superficial infections to life-threatening systemic infections. *C. albicans* can infect immunocompetent individuals, but these infections are especially serious in immunocompromised and elderly individuals [3]. *C. albicans* has also been associated with a number of gastrointestinal diseases including celiac disease and inflammatory bowel diseases (IBD), suggesting a role in their pathogenesis [2,4,5,6,7]. *C. albicans* would exacerbate inflammatory processes due to a sequence of events that perpetuate on each other: dysbiosis and low-level inflammation in the intestine fuels the growth of *C. albicans* while its overgrowth promotes further inflammation, exacerbating lesions and delaying healing [5,6,8]. This process would explain, at least in part, the link between *C. albicans* and these gastrointestinal diseases.

Several lines of evidence support the role of macrophages in inflammatory processes. Macrophages are known to orchestrate immune responses by initiating and resolving inflammatory signaling programs. Intestinal macrophages are an abundant cell population of the intestinal mucosa. They are essential for local homeostasis and to maintain the balance between microbiota and immune response [9], and are particularly recruited at the intestinal mucosa surface during *Candida* colonization [10]. Emerging evidence indicates that the state of macrophage polarization plays a critical role in the regulation of inflammatory processes and in the host susceptibility to infections. Macrophages release pro-inflammatory mediators involved in anti-infectious responses such as tumor necrosis factor alpha (TNF-α), interleukin (IL)-1, IL-6, IL-8, IL-12, cytokines, prostaglandin E2 (PGE2) and leukotriene B4 (LTB4), eicosanoids, and reactive oxygen or nitrogen species. By contrast, macrophages are critical in the resolution of inflammation and tissue repair, in particular by producing anti-inflammatory mediators as IL-10, TGF-β, prostaglandin D2 (PGD2) and lipoxin A4. In addition to their secretory properties, macrophages express receptors on their surface that are essential for yeast recognition and phagocytosis [11,12]. Among these receptors, C-type lectin receptors (CLR) as Dectin-1 and mannose receptor (MR) have been described to be essential in the direct recognition of *Candida* and in antifungal functions of macrophages [13,14,15,16].

Probiotics defined as live microorganisms that, when administered or consumed in adequate quantities, confer health benefits to the host have emerged as a new approach for the prevention and management of candidiasis. A number of in vitro studies have demonstrated that probiotics, particularly *Lactobacilli*, inhibit *C. albicans* growth and biofilm formation [17]. Numerous studies have been performed to substantiate the antifungal activity of probiotics in animals and humans, with oral cavity and urogenital tract as the major loci of investigation [17,18,19,20]. As regards the gastrointestinal tract, studies were mainly performed in immunocompromised children (preterm neonates) in which single or mixtures of probiotic strains reduced the incidence and intensity of enteric colonization by *Candida* spp. [17]. Furthermore, the administration of a mixture of *Lactobacillus helveticus* and *Lactobacillus rhamnosus* had beneficial effects, with a reduction of colonic damage, in patients with ulcerative colitis and in an experimental model of colitis in rats [21]. In addition, certain yeast probiotics belonging to the *Saccharomyces* and *Saccharomycopsis* genus have demonstrated beneficial effects in human and murine IBD models [22]. The effects of probiotics are well known to be strain-dependent and this is also the case in *Candida* infections [3,23].

Emerging evidence based on their ability to modulate cytokine release indicates that probiotics exhibit immunomodulatory properties both on the innate and adaptative immune systems [24,25,26]. Probiotics act on gut mucosal immunity by increasing the number of T and B lymphocytes, and macrophages [27]. Interestingly, probiotics play a dual role depending on the physiopathological context. Indeed, probiotics can be involved in immunostimulation by activating NK and Th1 cells to act against infection and cancer cells. Conversely, it has been shown in several inflammatory diseases that probiotics have immunoregulatory functions by inducing the differentiation of Tregs and the production of IL10 [24,28]. Consistently with their immunoregulatory activities, probiotics were also described to reduce the release of pro-inflammatory cytokines [24], supporting their use to control tissue inflammatory status.

Despite the growing knowledge with regard to the immunomodulatory properties of probiotics, little is known about how they control macrophage differentiation and the associated microbicidal functions. The objective of this study was to evaluate the effects of two lactobacilli strains, *Lactobacillus helveticus* LA401 and *Lactobacillus gasseri* LA806, in a murine model of oesophageal and gastrointestinal candidiasis (GIC).

We demonstrated that oral administration of *L. gasseri* LA806 alone or combined with *L. helveticus* LA401 in *Candida albicans* infected mice decrease gastrointestinal (GI) tract *C. albicans* burdens. Interestingly, the probiotic combination significantly modulates the composition of gut microbiota towards a protective profile and dampens inflammation and oxidative stress in the colon of mice with gastrointestinal candidiasis. Moreover, we showed that *L. helveticus* LA401 and/or *L. gasseri* LA806 orient macrophages towards a fungicidal phenotype characterized by an increase in CLR expression that participate in the defence against *C. albicans* while controlling inflammatory response. In conclusion, these data support the probiotic combination is a good candidate for managing candidiasis by inducing fungicidal functions in macrophages and preserving the GI integrity by modulating the microbiota and inflammation.

## 2. Materials and Methods

### 2.1. C. albicans and Bacterial Strains

The strain of *C. albicans* used throughout these experiments was provided by ATCC (ATCC^®^ MYA2876™), and was maintained on Sabouraud dextrose agar (SDA; Biorad, Hercules, CA, USA) plates containing gentamicin and chloramphenicol. Growth from an 18- to 24-h SDA culture of *C. albicans* was suspended in sterile saline solution (NaCl 0.9%) for mice administration or in culture medium for in vitro experiment [13,29].

*Lactobacillus helveticus* LA401 and *Lactobacillus gasseri* LA806 were provided by Genibio (Lorp-Sentaraille, Paris, France). The combination of the two strains is marketed under the name Lactibiane Cnd (PiLeJe Laboratoire, Paris, France).

### 2.2. Murine Model of Gastrointestinal Candidiasis

All mouse experiments were performed according to protocols approved by the institutional ethics committee (CEEA122) with permit number 5412-2016051917498658 in accordance with European legal and institutional guidelines (2010/63/UE) for the care and use of laboratory animals. Female C57BL/6 mice aged 8 weeks were purchased from Janvier Labs (Le Genest-Saint-Isle, France). Viable lyophilized bacteria were suspended in sterile saline solution. Each bacterial strain was administered orally at the dose of 1 × 10^9^ CFU once daily for 12 days before *C. albicans* administration and then 5 days after. The combination of the two strains contains 1 × 10^9^ CFU of each bacterial strain. Control groups only received the vehicle (saline solution). GIC was established by the intra-oesophageal administration of *C. albicans* at the amount of 50 × 10^6^ blastospores in sterile saline solution per mouse, as described previously [10,30]. In total, 10 mice were included in each experimental group. Stools were collected to quantify viable *C. albicans* at 3-, 4- and 5-days post *C. albicans* gavage. Then, 5 days after *C. albicans* administration, oesophagus, caecum and colon were aseptically removed to evaluate *C. albicans* colonization, and the microbiota and inflammatory status were evaluated in colon.

### 2.3. Quantification of the Number of Viable C. albicans in the Stools

Stools were collected daily from 2 days after gavage, weighed and mechanically homogenized in phosphate buffer saline (PBS). Serial dilutions of homogenates were plated onto CHROMAgar ^TM^ *Candida* plates (CHROMAgar, Paris France) for quantitative determination of the number of *C. albicans*. Plates were incubated at 37 °C for 24–48 h and the number of colonies was counted.

### 2.4. Quantification of C. albicans in the Gastrointestinal Tract and Microbiota Analysis Using Real-Time PCR

Oesophagus, caecum and colon from infected mice were crushed using lysing matrix tubes (MP Biomedicals, Illkirch-Graffenstaden, France). Tissue sample homogenates were resuspended in BLB lysis buffer (Roche, Meylan, France) for 20 min at room temperature and DNA was purified using High Pure PCR Template preparation kit (Roche). RT-quantitative PCR was performed with primers that amplify the genes encoding 16S rRNA from specific bacterial groups and the rDNA operon from *Candida* spp. on a Light Cycler 480 system using Light Cycler SYBR Green I Master (Roche). Primers are listed in Table 1. Serially diluted samples of genomic fungal DNA obtained from *C. albicans* cultures were used as external standards in each run. Cycle numbers of the logarithmic linear phase were plotted against the logarithm of the concentration of template DNA to evaluate the number of yeast cells present in each tissue sample homogenate.

For evaluation of mucosa-associated bacteria colonization, semi-quantitative PCR was performed on DNA isolated from colonic mucosa using primers listed in Table 1. Relative quantity was calculated and normalized to the amount of genomic β-actin. For amplicon detection, the Light Cycler DNA SYBR Green I kit was used as described by the manufacturer (Roche diagnostics, Meylan, France).

### 2.5. Gene Expression Analysis by Reverse Transcription and Real-Time PCR

mRNA from colonic tissues or macrophages were prepared and cDNA were synthetized according to the manufacturer’s recommendations (total RNA Minipreps super kit, BioBasic; Verso cDNA kit, Thermo Fisher Scientific). RT-PCR was performed on a Light Cycler 480 system with Light Cycler SYBR Green I Master Mix (Roche). Serially diluted samples of pooled cDNA were used as external standards in each run for the quantification. Primers, listed in Table 2, were designed with the software Primer 3. GAPDH was used as the housekeeping gene.

### 2.6. Preparation of Mouse Peritoneal Macrophages

Resident peritoneal cells were harvested by washing the peritoneal cavity of female C57BL/6 mice with sterile NaCl 0.9%. Cells were allowed to adhere for 2 h at 37 °C and 5% CO_2_ in Dulbecco’s modified Eagle’s medium (Invitrogen, Waltham, MA USA) supplemented with 5% heat-inactivated fetal calf serum. Non-adherent cells were then removed by washing with PBS without calcium and magnesium. Adherent macrophages were immediately stimulated or not with IFN-γ (40 UI/mL, Clinisciences, Nanterre, France) and LPS (10 ng/mL, Sigma, Lyon, France) for 24 h. Then, *Lactobacillus* strains were added at a ratio of 30 bacteria per macrophage for 4 h at 37°C before adding *Candida* to assess the killing, phagocytosis and binding ability of macrophages and their mRNA expression profile.

### 2.7. Killing Assay

Cells were allowed to interact for 2 h at 37 °C with *C. albicans* blastospores (at a ratio of 1 yeast per 3 macrophages) and unbound yeasts were removed by four washes with medium. Macrophages were then incubated at 37 °C for 4 h. After incubation, medium was removed and cells were lysed. Dilutions were inoculated in SDA plates and incubated as described above to determine the number of viable *C. albicans*. To evaluate superoxide anion (O_2_^−^) and nitric oxide (NO) cytotoxic activity, macrophages were incubated for 10 min before yeasts in presence of superoxide dismutase (30 IU/mL, Sigma) (scavenger for O_2_^−^) and L-NMMA (300 µM, Sigma) (inhibitor of NO production). Each assay was conducted in triplicates.

### 2.8. Binding and Phagocytosis Assays

Macrophages were co-cultured with *Lactobacillus* strains for 4 h and challenged with GFP-labelled yeasts at a ratio of 6 blastospores per macrophage. Binding was performed at 4 °C and phagocytosis at 37 °C with 5% CO_2_ and stopped after 1 h 30 min by washing with ice-cold PBS. The number of *C. albicans* bound or engulfed by macrophages was determined by fluorescence quantification using the fluorimetry-based approach (Envision, Perkin Elmer). Each assay was conducted in triplicates.

### 2.9. ROS Quantification

The oxygen-dependent respiratory burst of macrophages (ROS production) was measured by chemiluminescence in the presence of 5-amino-2,3-dihydro-1,4-phthalazinedione (luminol, Sigma) using a thermostatically (37 °C) controlled luminometer (Envision, Perkin Elmer). The generation of chemiluminescence was monitored continuously for 1 h 30 min with a challenge or not with *C. albicans* (yeast-to-macrophage ratio: 3:1). Each assay was conducted in triplicates. Statistical analysis was performed using the area under the curve expressed in counts × seconds.

### 2.10. Measurement of Nitrites (NO_2_^−^)

Peritoneal macrophages were treated with *Lactobacillus* strains for 10 h and challenged or not with *C. albicans* (yeast-to-macrophage ratio: 3:1). Culture supernatants of macrophages were incubated with equal volumes of Griess reagent, containing 1% sulfanilamide (Sigma) and 0.1% naphthylethylenediamine dihydrochloride (Sigma) in 2.5% phosphoric acid. After 30 min at room temperature, the absorbance was read at 550 nm and concentration was determined by comparison with standard solutions of sodium nitrite prepared in the same culture media. Each assay was conducted in triplicates.

### 2.11. Cytokine Measurement by ELISA

Cultured macrophages were treated or not with IFN-γ and LPS for 24 h, then with *Lactobacillus* strains for 10 h. IL12-p70, TNF-α, IL-1β, TGF-β and IL-10 production by macrophages was evaluated in the cell culture supernatant using the OptEIA ^TM^ Mouse Set (Becton–Dickinson France SA, Rungis, France), following the manufacturer’s instructions. Each assay was conducted in triplicates.

### 2.12. Statistical Analysis

GraphPad Prism (GraphPad Software, Inc., La Jolla, CA, USA) was used for graph preparation and statistical evaluation. Differences between groups were assessed using ANOVA, followed by nonparametric Mann-Whitney test. Data with *p*-value ≤ 0.05 were considered to be significant (* *p* ≤ 0.05, ** *p* ≤ 0.01, *** *p* ≤ 0.001, **** *p* ≤ 0.0001). Data represent mean values ± standard error of the mean (SEM).

## 3. Results

### 3.1. Lactobacillus gasseri LA806 Alone or Combined with Lactobacillus helveticus LA401 Effectively Reduces C. albicans Burden in Mice Gastrointestinal Tract

**Figure 1:**
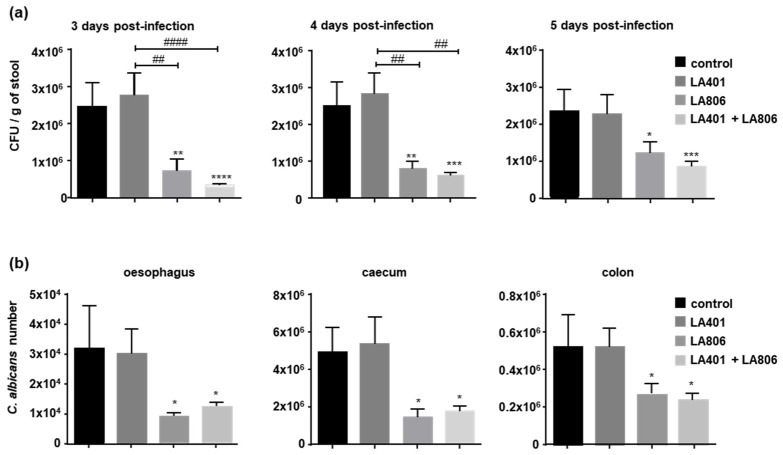
Effect of *Lactobacillus helveticus* LA401 and/or *Lactobacillus gasseri* LA806 oral administration on *Candida* burden. The bacterial strains (LA401, LA806 and combination of LA401 and LA806) were orally administered to mice (*n* = 10 per experimental group) once a day, for 12 days before yeast colonization. Mice were orally infected with *C. albicans* at 50 × 10^6^ blastospores per mouse. (**a**) Number of viable *C. albicans* were determined in stools collected at 3-, 4- and 5-days post *C. albicans* gavage. (**b**) On day 5 post *C. albicans* administration, mice were sacrificed and *C. albicans* colonization in the oesophagus, caecum and colon was assessed by quantitative RT-PCR. Data are presented as means ± SEM. * *p* ≤ 0.05, ** *p* ≤ 0.01, *** *p* ≤ 0.005, **** *p* ≤ 0.001 compared to control. ^##^ *p* ≤ 0.01, ^####^ *p* ≤ 0.001 compared to treatments.

To characterize the efficacy of probiotics on the gastrointestinal colonization by *Candida* we evaluated *Candida* burdens in stools, oesophagus, caecum and in colon after oral administration of *Lactobacillus helveticus* LA401 and *Lactobacillus gasseri* LA806 alone or in combination [10,30] (Figure 1). Although LA401 alone had no impact on the number of viable *C. albicans* in the faeces, LA806 alone or combined with LA401 significantly reduced that number from day 3 to day 5 post *C. albicans* administration with a greater antifungal activity when the two strains were conjointly administered (Figure 1a). In line with these observations, the number of *C. albicans* in the oesophagus, caecum and colon was significantly diminished in mice treated with LA806 alone or in combination with LA401 (Figure 1b), demonstrating that oral administration of LA806 alone or combined with LA401 favors the clearance of *C. albicans* throughout the GI tract.

### 3.2. Lactobacillus gasseri LA806 Alone or Combined with Lactobacillus helveticus LA401 Modulates Gut Microbiota

**Figure 2:**
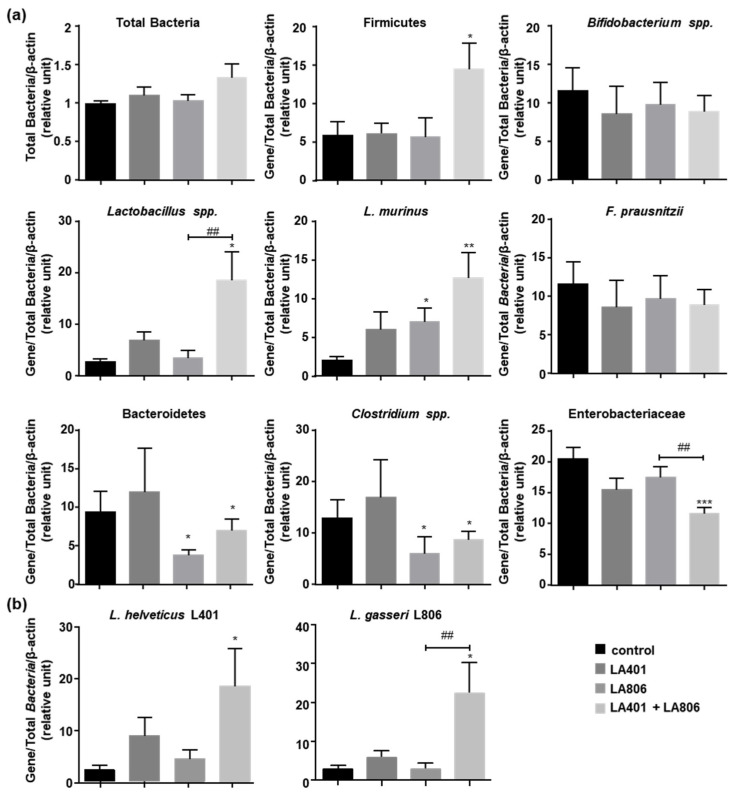
Impact of *Lactobacillus helveticus* LA401 and/or *Lactobacillus gasseri* LA806 oral administration on the colonic microbiota of *C. albicans*-infected mice. (**a**) The relative abundance of phyla, genus and bacteria species in the colonic mucosa of *Candida albicans*-infected mice treated with the bacterial strains (LA401, LA806 and the combination of LA401 and LA806) or not (control) was evaluated by RT-PCR. (**b**) Relative abundance of *L. helveticus* LA401 and *L. gasseri* LA806 in colonic mucosa of *C. albicans* infected-mice was evaluated by RT-PCR. Values were normalized to total bacteria and host β-actin. Primers are listed in Table 1. *n* = 10 per experimental group. Data are presented as means ± SEM. * *p* ≤ 0.05, ** *p* ≤ 0.01, *** *p* ≤ 0.005 compared to control. ^##^ *p* ≤ 0.01 compared to treatments.

We evaluated the composition of colonic mucosa-associated bacteria in mice subjected to GIC that were treated with LA401 and/or LA806. Although the strains alone or in combination did not change the total content of mucosa-associated bacteria (Figure 2a), they influenced the composition of certain phyla and bacterial species in the microbiota. While LA401 alone had no effect, LA806 alone significantly increased *Lactobacillus murinus*, which is a protective bacteria, and decreased Bacteroidetes and *Clostridium* spp. that contain a great number of pathobiontic bacteria (Figure 2a) [38,39]. It is interesting to note that the administration of LA401 and LA806 in combination strongly increased the content of Firmicutes, *Lactobacillus* spp. and *L. murinus*, described as beneficial and crucial bacteria for the health of intestinal mucosa. Consistently, LA401 and LA806 combination reduced the content of Bacteroidetes, *Clostridium* spp. and Enterobacteriaceae, which are often increased in dysbiosis (Figure 2a).

### 3.3. Lactobacillus helveticus LA401 and Lactobacillus gasseri LA806 Co-Administration Improves Their Respective Intestinal Colonization

To evaluate the attachment of LA401 and LA806 in the intestinal mucosa, we measured their abundance in the colon of *C. albicans* infected-mice that were orally administered with LA401 and/or LA806 (Figure 2b). When LA401 and LA806 were administered separately, there was no increase in their respective abundance, whereas when administered together their proportion significantly augmented. That demonstrates that the concomitant administration of the two strains improves their attachment to the intestinal mucosa revealing the benefit of using them in combination.

### 3.4. Lactobacillus helveticus LA401 and Lactobacillus gasseri LA806 Combination Dampens Inflammation and Oxidative Stress in the Colon of Mice with Gastrointestinal Candidiasis

**Figure 3:**
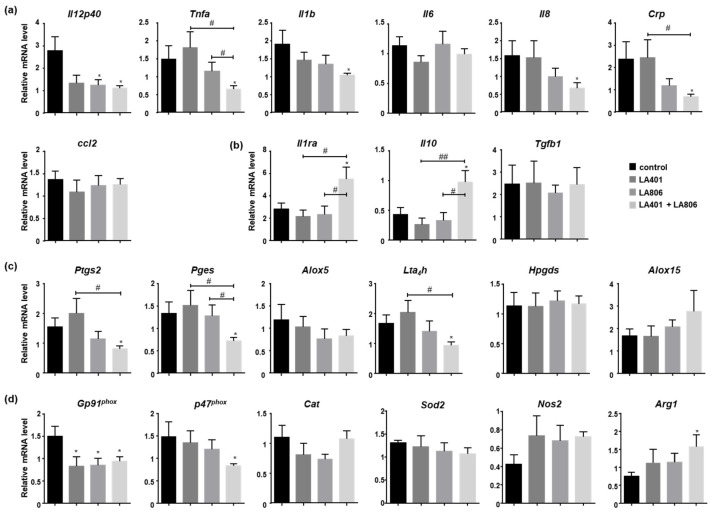
Influence of *Lactobacillus helveticus* LA401 and/or *Lactobacillus gasseri* LA806 oral administration on the colonic inflammatory and oxidative status of *C. albicans*-infected mice. LA401, LA806 alone or in combination were orally administered to mice for 12 days. After this treatment, mice were orally infected with *Candida albicans*. Mice were sacrificed 5 days later and total RNAs isolated from the colon were subjected to the RT-PCR analysis using specific primer sets for (**a**) pro-inflammatory cytokines and chemokines (*Il12p40*, *Tnfa*, *Il1b*, *Il6*, *Il8*, *Crp*, *Ccl2*), (**b**) for anti-inflammatory cytokines (*Il1ra*, *Il10*, *Tgfb1*), (**c**) for enzymes involved in the production of pro- or anti-inflammatory eicosanoids (*Ptgs2*, *Pges*, *Alox5*, *Lta_4_h*, *Hpgds*, *Alox15*) and (**d**) for enzymes involved in oxidative stress (*Gp91^phox^*, *p47^phox^*, *Cat*, *Sod2*, *Nos2*, *Arg1).* Primers are listed in Table 2. *n* = 10 per experimental group. Data are presented as means ± SEM. * *p* ≤ 0.05 compared to control. ^#^ *p* ≤ 0.05, ^##^ *p* ≤ 0.01 compared to treatments.

To investigate the effect of LA401 and/or LA806 on colonic inflammation in mice infected with *C. albicans*, we assessed the colonic expression of pro- and anti-inflammatory markers. While the administration of the strains separately did not significantly alter the expression of pro-inflammatory genes, with the exception of *Il12*, which was reduced with LA806 alone, LA401 and LA806 combination significantly decreased the expression of *Il12*, *Tnf-α*, *Il1b*, *Il8* and *Crp* inflammatory markers (Figure 3a). These findings were corroborated by the reciprocal increase in the expression of *Il1ra* and *Il10* anti-inflammatory markers in colonic tissues of *C. albicans*-infected mice that received the LA401 and LA806 combination (Figure 3b).

Consistent with the decrease in pro-inflammatory markers, the LA401 and LA806 combination also decreased the mRNA expression of enzymes involved in the synthesis of pro-inflammatory eicosanoids, *Ptgs2* [Cyclooxygenase-2], *Pges* [Prostaglandin E synthase], *Alox5* [5-Lipoxygenase] and *Lta4h* [LTB4 hydrolase critical to produce the pro-inflammatory mediator LTB4]) (Figure 3c). The mRNA expression of enzymes involved in the production of anti-inflammatory eicosanoids (*Hpgds* [Prostaglandin D synthase], *Alox15* [12/15-Lipoxygenase]) was not affected by the administration of LA401 and LA806 (Figure 3c).

Regarding the oxidative stress status of colon, the mRNA expression of *Gp91^phox^ and p47^phox^*, cytosolic subunits of the NADPH oxidase complex whose activation is essential for reactive oxygen species (ROS) release, were downregulated in response to LA401 and LA806 combination (Figure 3d). The expression of *Gp91^phox^* was also decreased when the strains were used individually. Moreover, the administration of LA401 and/or LA806 did not change the expression of inducible nitric oxide synthase (*Nos2*) and antioxidant enzymes, catalase-1 (*Cat*) and superoxide dismutase (*Sod2*) (Figure 3d). In accordance with this reduced oxidative status, the LA401 and LA806 combination has shifted the balance between *Nos2* (inducible nitric oxide synthase) and *Arg1* (arginase-1) towards the expression of arginase-1 (Figure 3d). Altogether these data highlight the anti-inflammatory and anti-oxidant potential of the LA401 and LA806 combination in the colon of mice infected with *C. albicans*.

### 3.5. Lactobacillus helveticus LA401 and/or Lactobacillus gasseri LA806 Improve the Fungicidal Properties of Macrophages While Controlling Their Inflammatory Status

**Figure 4:**
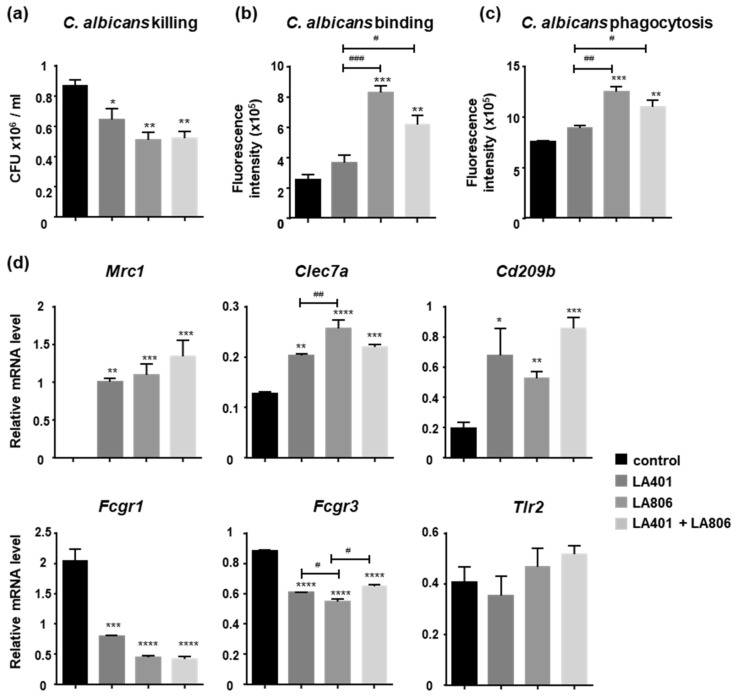
In vitro modulation of the anti-fungal activity of macrophages by *Lactobacillus helveticus* LA401 and/or *Lactobacillus gasseri* LA806. (**a**) Killing assay of murine peritoneal macrophages treated or not with LA401 and LA806 alone or in combination incubated with *Candida albicans*. (**b**) Binding and (**c**) phagocytosis of *C. albicans* by murine peritoneal macrophages treated or not with LA401 and LA806 alone or in combination. (**d**) Gene expression analysis of Pattern Recognition Receptors by IFN-γ and LPS-activated macrophages in response to probiotic treatment (*Mrc1*, *Clec7a*, *Cd209b*, *Tlr2*) and Fcγ receptors (*Fcgr1*, *Fcgr3*). Results are represented as means ± SEM of triplicates. * *p* ≤ 0.05, ** *p* ≤ 0.01, *** *p* ≤ 0.005, **** *p* ≤ 0.001 compared to control. ^#^ *p* ≤ 0.05, ^##^ *p* ≤0.01, ^###^ *p* ≤ 0.005 compared to treatments.

Previous work from our laboratory established the importance of fungicidal functions of macrophages in the outcome of GIC [10]. To investigate whether the treatment of macrophages with LA401 and/or LA806 can activate their fungicidal activity, we evaluated the ability of LA401 and/or LA806-treated macrophages to kill yeasts in vitro. Macrophages treated with LA401 and LA806 alone or in combination reduced the number of *C. albicans* more effectively than untreated macrophages (Figure 4a). Thus, treatment of macrophages with the two separate or combined strains improved their ability to kill *C. albicans*, demonstrating the potential of these probiotics to induce macrophage-intrinsic antifungal activity.

Supporting this observation, macrophages treated with LA806 alone or with the combination were more effective in binding and engulfing *C. albicans* (Figure 4b,c). Consistently with the involvement of mannose receptor, dectin-1 and SIGN-R1 C-type lectin receptors (CLRs) in the recognition of *Candida* and in the antifungal functions of macrophages [13,14], LA401 and/or LA806-treated macrophages displayed an upregulation of *Mrc1* [mannose receptor], *Clec7a* [dectin-1] and *Cd209b* [SIGN-R1] (Figure 4d). The induction of CLR expression by LA401 and/or LA806 was mirrored by a downregulation of mRNA levels of Fcγ receptors (*Fcgr1* [CD64] and *Fcgr3* [CD16]) on macrophages (Figure 4d). The mRNA expression of *Tlr2* on macrophages was not changed by probiotic treatment. These data provide evidence that LA401 and/or LA806 improve the fungicidal properties of macrophages through their ability to modulate CLR expression on macrophages.

**Figure 5:**
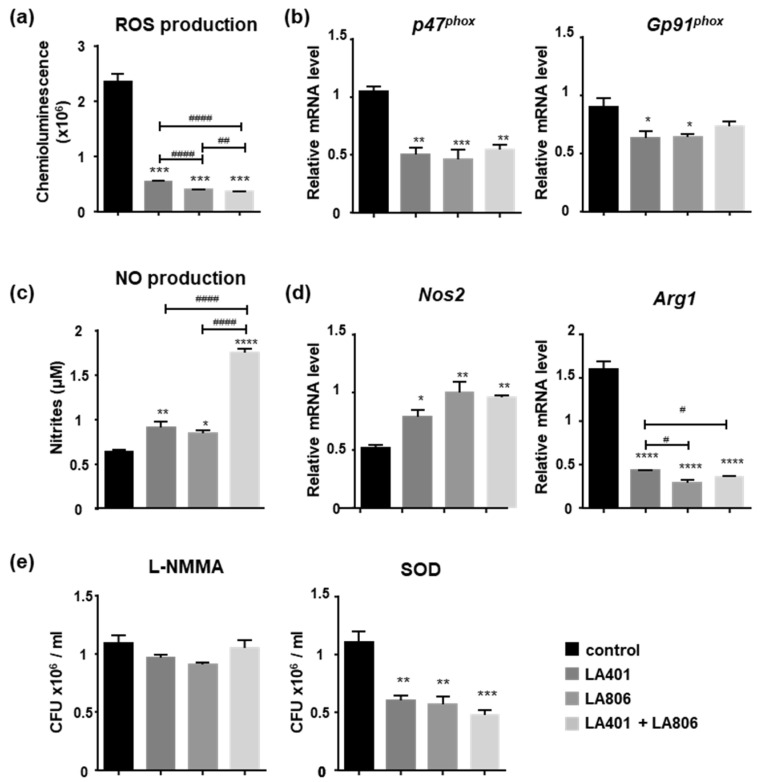
In vitro modulation of the oxidative fungicidal properties of macrophages by *Lactobacillus helveticus* LA401 and/or *Lactobacillus gasseri* LA806. (**a**) Reactive oxygen species (ROS) production by macrophages treated or not with LA401 and LA806 alone or in combination in response to *Candida albicans* challenge. (**b**) Gene expression analysis of enzymes involved in oxidative stress (*p47^phox^*, *Gp91^phox^*) by IFN-γ and LPS-activated macrophages in response to stimulation with LA806 and LA401 by qRT-PCR. (**c**) NO release by macrophages treated or not with LA401 and LA806 alone or in combination in response to *C. albicans* challenge. (**d**) Gene expression analysis of *Nos2* and *Arg1* by IFN-γ and LPS-activated macrophages treated or not with LA401 and LA806 alone or in combination. (**e**) Killing assay of macrophages treated or not with LA401 and LA806 alone or in combination incubated with *C. albicans* in the presence of an inhibitor of NO production (L-NMMA) or a scavenger for O_2_^−^ (SOD, superoxide dismutase). Results correspond to mean ± SEM of triplicates. * *p* ≤ 0.05, ** *p* ≤ 0.01, *** *p* ≤ 0.005, **** *p* ≤ 0.001 compared to untreated-macrophages. ^#^ *p* ≤ 0.05, ^##^ *p* ≤ 0.01, ^####^ *p* ≤ 0.001 compared to macrophages treated with probiotics.

Among their critical microbicidal functions, macrophages can also release large amounts of highly toxic molecules, such as reactive oxygen and nitrogen intermediates [30]. Surprisingly, LA401 and/or LA806 strongly decreased ROS production in macrophages in response to *C. albicans*, suggesting that ROS release is not involved in the fungicidal activity of probiotic-activated macrophages (Figure 5a). This decrease in ROS production was supported by a significant diminution of *p47^phox^* and *Gp91^phox^* expression, cytosolic subunits of the NADPH oxidase complex whose activation is essential for ROS release (Figure 5b). Inversely to their effect on ROS production, LA401 and LA806 alone, and more robustly, the combination of the two strains promoted NO release by macrophages in response to *C. albicans* challenge (Figure 5c). This observation was associated with an increase in the expression of the inducible NO Synthase (*Nos2*) and a downregulation of the expression of arginase-1 (*Arg1*) in LA401 and/or LA806-treated macrophages (Figure 5d).

To determine the involvement of ROS and NO in the fungicidal function of LA401 and/or LA806-treated macrophages, we evaluated the ability of LA401 and/or LA806-treated macrophages to kill *C. albicans* in the presence of L-NMMA (a specific competitor of L-arginine) or SOD (a specific inhibitor of superoxide anion production). We observed that NO release is essential for the fungicidal activity of LA806 and/or LA401-treated macrophages, since the inhibition of NO production by L-NMMA totally abolished their fungicidal effect (Figure 5e). In contrast, the sustained killing activity of LA401 and/or LA806-treated macrophages in the presence of SOD confirmed that ROS production is not involved in the fungicidal activity of probiotic activated-macrophages (Figure 5e).

**Figure 6:**
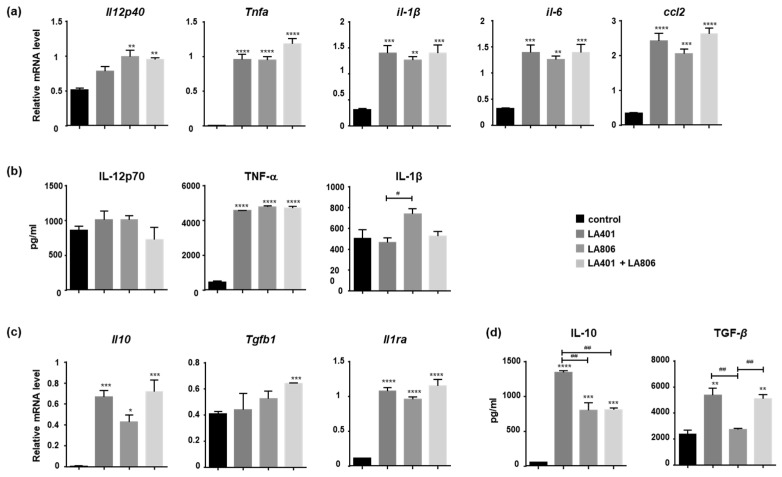
In vitro modulation of pro- and anti-inflammatory cytokine release of macrophages by *Lactobacillus helveticus* LA401 and/or *Lactobacillus gasseri* LA806. (**a**) mRNA and (**b**) protein levels of pro-inflammatory cytokines (*Il12p40*, *Tnfa*, *Il1b*, *Il6* and *Ccl2;* IL-12p70, TNF-α, IL-1β); (**c**) mRNA and (**d**) protein levels of anti-inflammatory cytokines mRNA (*Il10*, *Tgfb1* and *Il1ra*; IL-10, TGF-β) of IFN-γ and LPS-activated macrophages treated with LA401 and LA806 strains alone or in combination. Results are presented as means ± SEM of triplicates. * *p* ≤ 0.05, ** *p* ≤ 0.01, *** *p* ≤ 0.005, **** *p* ≤ 0.001 compared to control. ^#^ *p* ≤ 0.05, ^##^ *p* ≤ 0.01 compared to treatments.

Given the major regulatory role of cytokines in the immune response against fungal pathogens [40], we evaluated the ability of LA401 and/or LA806 strains to modulate the expression of pro- and anti-inflammatory cytokines and chemokines in IFN-γ/LPS-activated macrophages. LA401 and/or LA806 increased the mRNA and protein expression of the pro-inflammatory cytokines IL-12, TNF-α, IL-1β and IL-6, as well as the chemokine CCL2 (Figure 6a,b), suggesting that LA401 and/or LA806 promote antifungal host defense through their ability to modulate the release of pro-inflammatory mediators involved in the protection against fungal pathogens by macrophages. Interestingly, the increase in pro-inflammatory markers induced by LA401 and/or LA806 was accompanied with the induction of IL-10 and TGF-β, and *Il-1ra* anti-inflammatory markers (Figure 6c,d). Taken together, these data demonstrate that LA401 and/or LA806 orient macrophages towards a fungicidal phenotype that participate in defense against fungal agents while controlling inflammatory response.

## 4. Discussion

*Candida* commonly colonizes the human GI tract as a commensal component of the resident microbiota. However, high level of *Candida* colonization is associated with several digestive diseases and appears to exacerbate inflammation [5]. Previous studies have reported that probiotics are potentially promising for the prevention or treatment of *Candida* infections [17,40] and that different *Lactobacillus* species can affect the immunomodulatory ability of various cellular components of the mucosal immune system [41].

In the present study, we observed that the oral administration of *Lactobacillus gasseri* LA806 alone or combined with *Lactobacillus helveticus* LA401 effectively reduced *C. albicans* number in the GI tract in mice. These two lactobacilli strains were selected based on preliminary in vitro assays showing that these strains were able to inhibit the growth of *C. albicans* and their adhesion on Caco-2 cells (internal data). Previous data demonstrated that *Lactobacillus helveticus* HY7801 ameliorated vulvovaginal candidiasis in mice by inhibiting fungal growth and NF-*k*B activation [42]. Consistently, it has been shown that *Lactobacillus gasseri* strains isolated from vaginal swabs of healthy women had anti-*Candida* activity [43] and that *Lactobacillus gasseri* OLL2716 presented anti-inflammatory properties [44]. All these data supported potential anti-fungal and anti-inflammatory activities of *L. helveticus* or *L. gasseri* strains.

In line with the mucosal bacterial dysbiosis induced by *Candida* colonization and the modulation of gut microbiota composition by probiotics [45,46], we investigated the composition of colonic mucosa-associated bacteria in mice subjected to GI candidiasis that were treated with LA401 and/or LA806. While different individual *L. helveticus* strains were shown to alleviate the decrease of *Lactobacillus* and Firmicutes induced by *Candida* infection and to decrease the Enterobacteriaceae [43,47], in our study, *L. helveticus* LA401 had no effect. Only the administration of the combination of LA401 and LA806 increased the content of Firmicutes, *Lactobacillus* spp. And *L. murinus*, and reduced the content of Bacteroidetes, *Clostridium* spp. and Enterobacteriaceae, which are often increased in dysbiosis [48]. The orientation of the composition of gut microbiota towards a protective profile only after administration of the two strains suggests a better attachment to the intestinal mucosa when they are combined.

In line with the orientation of the colonic microflora towards protective bacteria and the decrease of *C. albicans* colonization by the combination of LA401 and LA806, the probiotic combination also dampened colonic inflammation. These results were consistent with the potential for *Candida* and *Enterobacteriaceae* to induce intestinal inflammation [5,48]. Remarkably, the two bacterial strains conjointly administered reduced the colonic expression of several pro-inflammatory markers such as cytokines and enzymes involved in the synthesis of pro-inflammatory eicosanoids, and reciprocally increased the expression of anti-inflammatory markers. Our data are in line with those of Kimoto-Nira et al. who demonstrated the ability of *Lactobacillus* to reduce the production of the pro-inflammatory eicosanoid LTB4 [49] and with previous studies showing that certain *L. gasseri* and *L. helveticus* strains have a high potential for the management of inflammatory pathologies and for inhibiting NF-*k*B activation [43,50,51]. Associated with their anti-inflammatory effects, the LA401 and LA806 combination also had an anti-oxidant potential. In line with this finding, the protective effects of different *L. gasseri* and *L. helveticus* strains against oxidative stress were previously established [52,53].

Previous studies have shown that different *Lactobacillus* species can affect the immunomodulatory ability of various cellular components of the innate and mucosal immune systems [42]. Anti-inflammatory activities have been reported; it was shown that *Lactobacillus* species can induce Treg differentiation and suppress the development of dermatitis, asthma and IBD [38,54,55]. As macrophages play a direct role in fungicidal activity through their ability to phagocytose yeasts and to release large amounts of highly toxic molecules, such as reactive oxygen intermediates and reactive nitrogen intermediates [14], we evaluated the potential of the two strains on macrophage-intrinsic antifungal activity. Then, we demonstrated that LA401 and LA806 in combination increased the ability of macrophages to bind, engulf and eliminate *C. albicans*. In relation with this result, the strains up-regulated Mannose receptor, Dectin-1 and SIGNR1 expression on macrophages, receptors previously described as being involved in yeast recognition, phagocytosis and clearance [13,14,30,56]. Conversely, the downregulation of the expression of Fcγ receptors Fc-RI, III (CD64 and CD16) on macrophages during treatment with LA401 and LA806 support the fact that these probiotics promote fungal recognition mediated by CLRs. CLR-dependent microbial recognition, which does not require the opsonization of pathogens, is particularly interesting for immunocompromised hosts [30]. This is best supported by the impact of many probiotic strains on phagocytosis [57], and by the increase of MR and TLR2 on macrophages and dendritic cells after oral *L. casei* administration [58].

Consistent with the anti-fungal properties of macrophages, the expression of pro-inflammatory cytokines, such as IL-12, TNF-α, IL-1β, and IL-6, and the CCL2 chemokine were increased in macrophages treated with the combination of LA401 and LA806. Simultaneously, the macrophages treated with probiotics released also large amount of IL-10 and TGF-β, and strongly expressed the *Il-1ra* anti-inflammatory marker. Thus, these results show that LA401 and LA806 strains oriented the macrophages towards both a fungicidal pro-inflammatory phenotype that participates in the defense against fungi and a pro-resolutive phenotype that controls the deleterious inflammatory response. This dual phenotype was reinforced by a strong expression of CLRs, receptors preferentially expressed on pro-resolutive and anti-inflammatory macrophages, but which was also coupled to pro-inflammatory signaling pathways in response to pathogens [11,13,14]. In addition to the increased anti-fungal activity of LA401- and LA806-treated macrophages through CLR recognition and cytokine release, this study also showed the essential involvement of NO release for the fungicidal activity of LA806- and LA401-treated macrophages.

## 5. Conclusions

In conclusion, *L. helveticus* LA401 together with *L. gasseri* LA806 have a protective role in GI candidiasis and more specifically in limiting the colonization of the gastrointestinal tract by *C. albicans*. Here we provide evidence that these two strains significantly promote the intrinsic antifungal activities of macrophages. Moreover, these strains and in particular their combination can modulate the composition of the mucosa-associated microbiota by favoring protective microbiota and consequently attenuate the inflammatory status of the colon. Our findings suggest that the use of the LA401 and LA806 combination might be a promising strategy to manage GI candidiasis and the inflammation it causes by inducing the fungicidal functions of macrophages.

## Figures and Tables

**Table 1:** Primers used for gut microbiota analysis.

Gene	Universal Name		5’-3’ Sequence
*Candida* spp. [31]	sense		TCGCATCGATGAAGAACGCAGC
	antisense		TCTTTTCCTCCGCTTATTGATATGC
*Clostridium* spp. [32]	sense		CGGTACCTGACTAAGAAGC
	antisense		AGTTTYATTCTTGCGAACG
*Bifidobacterium* spp. [32]	sense		GGGTGGTAATGCCGGATG
	antisense		TAAGCGATGGACTTTCACACC
*Lactobacillus* spp. [32]	sense		AGCAGTAGGGAATCTTCCA
	antisense		CACCGCTACACATGGAG
Total bacteria [33]	sense	Eub338F	ACTCCTACGGGAGGCAGCAG
	antisense	Eub518R	ATTACCGCGGCTGCTGG
Bacteroidetes [33]	sense	Bact934F	GGARCATGTGGTTTAATTCGATGAT
	antisense	Bact1060R	AGCTGACGACAACCATGCAG
Firmicutes [33]	sense	Firm934F	GGAGYATGTGGTTTAATTCGAAGCA
	antisense	Firm1060R	AGCTGACGACAACCATGCAC
Enterobacteriaceae [34]	sense	Uni515F	GTGCCAGCMGCCGCGGTAA
	antisense	Ent826R	GCCTCAAGGGCACAACCTCCAAG
*Faecalibacterium prausnitzii* [35]	sense	Fprau223F	GATGGCCTCGCGTCCGATTAG
	antisense	Fprau420R	CCGAAGACCTTCTTCCTCC
*Lactobacillus murinus/animalis* [36]	sense		TCGAACGAAACTTCTTTATCACC
	antisense		ATGACCCAGATCATGTTTGA
*Lactobacillus helveticus*	sense		ACCTGCCCCATAGTCTAGGA
	antisense		ACGCCGCCTTTTATAAGCTG
*Lactobacillus gasseri*	sense		AGACATGCGTCTAGTGTTGTT
	antisense		TGGGTAACCTGCCCAAGAGA
Genomic actin [37]	sense		ATGACCCAGATCATGTTTGA
	antisense		TACGACCAGAGGCATACAG
Fungi [38]	sense	ITS1-2 F	CTTGGTCATTTAGAGGAAGTAA
	antisense	ITS1-2 R	GCTGCGTTCTTCATCGATGC

**Table 2:** Primer sequences used in qRT-PCR.

Gene	5’-3’Sequence	Sequence	Function
*Alox15*	sense	GTTCAGGAACCACAGGGAGG	12/15-Lipoxygenase
	antisense	GTCAGAGATACTGGTCGCCG	enzyme involved in the synthesis of anti-inflammatory eicosanoids
*Alox5*	sense	AGAGCGGCAGCTCAGTTTAG	5-Lipoxygenase
	antisense	GGAACTGGTGTGTACAGGGG	enzyme involved in the synthesis of pro-inflammatory eicosanoids
*Arg1*	sense	CGTGTACATTGGCTTGCGAG	Arginase-1/anti-inflammatory marker
	antisense	TCGGCCTTTTCTTCCTTCCC	/by degrading arginine, deprives NOS2 of its substrate
*Cat*	sense	ACATGGTCTGGGACTTCTGG	Catalase-1
	antisense	CAAGTTTTTGATGCCCTGGT	antioxidant enzyme
*Ccl2*	sense	AGGTCCCTGTCATGCTTCTG	pro-inflammatory chemokine
	antisense	TCTGGACCCATTCCTTCTTG	recruit monocytes to the site of inflammation
*Cd209b*	sense	GGCACGAAAGTGAGGCACAT	SIGNR1/C-type lectin receptor
	antisense	AGCTCATCTCCGCTCCTACCT	macrophage surface receptor
*Clec7a*	sense	CCTCCAAGGCATCCCAAACT	Dectin-1/C-type lectin receptor
	antisense	TAGCTGGGAGCAGTGTCTCT	macrophage surface receptor
*Crp*	sense	CGCAGCTTCAGTGTCTTCTC	C reactive protein
	antisense	AGATGTGTGTTGGAGCCTCA	inflammatory marker
*Fcgr3*	sense	TGTTTGCTTTTGCAGACAGG	CD16 Fcγ receptors
	antisense	TGCTCCATTTGACACCGATA	macrophage surface receptor
*Fcgr1*	sense	GTTATTGCCACCAAGGCTGT	CD64 Fcγ receptors
	antisense	ACCTGTATTCGTCACTGTCC	macrophage surface receptor
*Gapdh*	sense	ACACATTGGGGGTAGGAACA	housekeeping
	antisense	AACTTTGGCATTGTGGAAGG	
*Gp91phox*	sense	ACTGCGGAGAGTTTGGAAGA	cytosolic subunit of the NADPH oxidase complex/reactive oxygen species release
	antisense	GGTGATGACCACCTTTTGCT	
*Hpgds*	sense	GGACACGCTGGATGACTTCA	Prostaglandin D synthase
	antisense	TCCCAGTAGAAGTCTGCCCA	enzyme involved in the synthesis of anti-inflammatory eicosanoids
*Il10*	sense	AGGCGCTGTCATCGATTTCT	anti-inflammatory cytokine
	antisense	GCTCCACTGCCTTGCTCTTA	
*Il12p40*	sense	AGGTCACACTGGACCAAAGG	pro-inflammatory cytokine
	antisense	TGGTTTGATGATGTCCCTGA	
*Il1ra*	sense	GGCCTAGGTGTCTTCTGCTC	Interleukin-1 receptor antagonist
	antisense	GTAAGGGAGTCACTTGGGGC	anti-inflammatory marker
*Il1b*	sense	CAACCAACAAGTGATATTCTCGATG	pro-inflammatory cytokine
	antisense	GATCCACACTCTCCAGCTGCA	
*Il6*	sense	GAGGATACCACTCCCAACAGACC	pro-inflammatory cytokine
	antisense	AAGTGCATCATCGTTGTTCATACA	
*Il8*	sense	TCCCTTGTGGAGGCTAGAGA	pro-inflammatory cytokine
	antisense	AGGCACAGGTAGGATCC	
*Lta4h*	sense	GTTGACAGCTGAACCCCAGT	LTB4 hydrolase critical to produce the pro-inflammatory mediator LTB4
	antisense	CGTGCCCTTAGTTCCACATT	
*Mrc1*	sense	GGGTTCACCTGGAGTGATGG	Mannose receptor/C-type lectin receptor
	antisense	ATGCCAGGGTCACCTTTCAG	macrophage surface receptor
*Nos2*	sense	TCCTGGACATTACGACCCCT	Inducible Nitric oxide synthase
	antisense	ACAAGGCCTCCAATCTCTGC	pro-inflammatory marker
*Pges*	sense	CCTAGGCTTCAGCCTCACAC	Prostaglandin E synthase
	antisense	CAGCCTATTGTTCAGCGACA	enzyme involved in the synthesis of pro-inflammatory eicosanoids
*Ptgs2*	sense	AGAAGGAAATGGCTGCAGAA	Cyclooxygenase-2
	antisense	GCTCGGCTTCCAGTATTGAG	enzyme involved in the synthesis of pro/anti-inflammatory eicosanoids
*p47phox (Ncf1)*	sense	AGTGATGCGGAGACTTTGCT	cytosolic subunit of the NADPH oxidase complex/reactive oxygen species release
	antisense	ACCGGAGTTACAGGCAAATG	
*Sod2*	sense	GCCCCCTGAGTTGTTGAATA	Superoxide dismutase-2
	antisense	AGACAGGCAAGGCTCTACCA	antioxidant enzyme
*Tgfb1*	sense	AGGTTGGCATTCCACTTCAC	anti-inflammatory cytokine
	antisense	AGGGGCCTCTAAGAGCAGTC	
*Tlr2*	sense	TGCTTTCCTGCTGGAGATTT	Toll like receptor-2
	antisense	TGTAACGCAACAGCTTCAGG	macrophage surface receptor
*Tnfa*	sense	AGCCCCCAGTCTGTATCCTT	pro-inflammatory cytokine
	antisense	CTCCCTTTGCAGAACTCAGG	

## Data Availability

Not applicable.
